# Current State of Surgical Management of Pancreatic Cancer

**DOI:** 10.3390/cancers3011253

**Published:** 2011-03-10

**Authors:** Thilo Hackert, Markus W. Büchler, Jens Werner

**Affiliations:** Department of General, Visceral and Transplantation Surgery, University of Heidelberg, Im Neuenheimer Feld 110, 69120 Heidelberg, Germany; E-Mails: markus_buechler@med.uni-heidelberg.de (M.W.B.); jens_werner@med.uni-heidelberg.de (J.W.)

**Keywords:** pancreatic cancer, surgery, standard resection, extended approach

## Abstract

Pancreatic cancer is still associated with a poor prognosis and remains—as the fourth leading cause of cancer related mortality—a therapeutic challenge. Overall long-term survival is about 1–5%, and in only 10–20% of pancreatic cancer patients is potentially curative surgery possible, increasing five-year survival rates to approximately 20–25%. Pancreatic surgery is a technically challenging procedure and has significantly changed during the past decades with regard to technical aspects as well as perioperative care. Standardized resections can be carried out with low morbidity and mortality below 5% in high volume institutions. Furthermore, there is growing evidence that also more extended resections including multivisceral approaches, vessel reconstructions or surgery for tumor recurrence can be carried out safely with favorable outcomes. The impact of adjuvant treatment, especially chemotherapy, has increased dramatically within recent years, leading to significantly improved postoperative survival, making pancreatic cancer therapy an interdisciplinary approach to achieve best results.

## 1. Introduction

Management of pancreatic cancer (PDAC) is an interdisciplinary challenge as this tumor entity is still characterized by a poor prognosis, with long-term survival of only 1–5% [1]. From the oncological perspective, pancreatic cancer represents the fourth leading cause for cancer related mortality in the Western world with more than 100,000 deaths in Europe and the USA per year [2]. A major problem is early detection since 80–90% of pancreatic cancers are locally or systemically advanced at the time of diagnosis. However, in patients who are suitable for resection, five-year survival rates of 25% are possible [3], which underlines that surgery offers the only chance of cure and long-term survival. Yet, surgical therapy has to be embedded in an oncological concept of adjuvant treatment as postoperative chemotherapy is a key factor to further improve patient survival [4,5]. Numerous ongoing studies on new therapeutic agents like antibodies, antimetabolites and supportive agents reflect the current scientific and clinical struggle to achieve a better outcome of pancreatic cancer patients in the future [6]. Despite new chemotherapeutical or targeted substances, long-term survival needs to be based on an initial tumor resection [3,7]. Today, standardization of surgical procedures and centralization of pancreatic surgery in high volume institutions guarantees the best patient care and mortality rates below 5% [8-10]. This review summarizes the current state of pancreatic surgery for malignant pathologies with focus on standard resections, the impact of lymphadenectomy, as well as extended indications, namely resection of vascular structures, adjacent organs and tumor recurrences.

## 2. Standard Resections

Standard resections include partial pancreatico-duodenectomy with distal stomach resection or— recently accepted as the preferable procedure—preservation of the pylorus for tumors in the head of the pancreas, distal pancreatectomy for tumors of the corpus and tail as well as total pancreatectomy for more extended tumors or intraductal papillary mucinous neoplasias (IPMN) if necessary. Venous resections including the portal and superior mesenteric vein during these procedures are well-accepted as described below.

### 2.1. Partial Pancreatico-Duodenectomy

**Figure 1.**
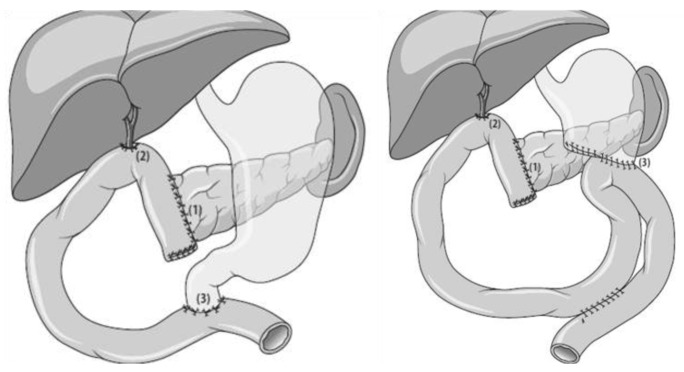
Pylorus-preserving (left) and classical (right) pancreatico-duodenectomy. End-to-side pancreatico-jejunostomy (1), end-to-side hepato-jejunostomy (2) and end-to-side duodeno- or gastro-jejunostomy (3), respectively.

Partial pancreatico-duodenectomy with resection of the distal stomach (Whipple resection) is the historical standard procedure for tumors of the pancreatic head. During the last two decades, preservation of the pylorus has been widely accepted, as proven to be equally effective compared to the classical pancreatico-duodenectomy with regard to tumor recurrence and long-term survival [11], Figure 1. A recent meta-analysis has confirmed these findings and, furthermore, has shown that preservation of the pylorus shortens operation time and reduces blood loss [12], Table 1. Therefore, the classical Whipple procedure should only be performed in situations where tumor spread towards the stomach cannot be ruled out, lymph node metastases are suspected in this area or distal stomach perfusion is critical, e.g., due to dissection of venous vessels. A crucial step during reconstruction after partial pancreatico-duodenectomy is the creation of the pancreatic anastomosis. A large number of variations including pancreatico-gastrostomy and pancreatico-jejunostomy with or without internal stent placement have been introduced and are currently used worldwide. The most important aspect of this anastomosis is technical standardization to achieve low fistula rates and avoid further consequent complications. The consensus paper of the International Study Group Pancreatic Fistula in 2005 [13] has defined postoperative fistula as drainage fluid on or after day 3 postoperatively with an amylase content of at least three-times that of serum amylase activity. The additional clinical grading of A-C reflects the severity and potential danger for the patient and makes study results on various anastomosis techniques comparable in a standardized way [14,15]. Surgical techniques with low fistula rates such as the binding anastomosis [16] or the duct-to-mucosa suture with internal stenting [17] have been reported and are currently used. Regardless which kind of anastomosis is performed, insufficiency rates of less than 3.5% should be achieved [18]. Bile-duct reconstruction should be standardized as well to avoid insufficiency and postoperative bile collection. Leakages of the hepatico-jejunostomy occur less frequent than pancreatic fistulas, but can also cause severe and long-lasting complications. The end-to side duodeno-jejunostomy or gastro-jejunostomy completes the reconstruction. Several studies and a current metaanalysis have shown that antecolic reconstruction should be preferred to avoid delayed gastric emptying [19,20]. This observation can be explained by the interposition of omental tissue and the transverse colon between the pancreatic anastomosis and the stomach which protects the anastomosis from any inflammatory or chemical irritation by the pancreatic anastomosis which may cause gastric dysfunction and emptying delay.

### 2.2. Distal Pancreatectomy

Distal pancreatectomy is the standard procedure for tumors in the body or tail of the pancreas and usually includes splenectomy. Tumors located above or left of the superior mesenteric vein are suitable for this procedure. Dissection of the pancreas is usually performed above the vein after tunneling. This can be done at the beginning of the resection as proposed by Strasberg *et al.* [21]. During this procedure, the splenic vessels can be divided early, which offers good bleeding control and facilitates lymph node dissection along the celiac axis and the left aspect of the superior mesenteric artery. Proceeding to the left side, the specimen can be removed en bloc with the spleen. A special aspect in this procedure is the dorsal resection plane that needs to be defined by the surgeon either on the level of Gerota's fascia, that may be removed, or on the left kidney itself. The latter procedure is useful in cases of suspected tumor growth towards the left adrenal gland, which is not uncommon in tumors of the pancreatic tail [22]. All soft and lymphatic tissue along the left kidney vein can be removed under good visibility by this approach. Furthermore, the intention of an early division of the splenic vessels seems reasonable from the oncological point of view to minimize venous drainage from the pancreatic body and tail during surgical manipulation.

Transection of the pancreas itself is not standardized and can be done sharply, by electrocautery or by a stapling device. To date, there is evidence to support either procedure. In case of sharp dissection, the remnant is closed by sutures with a moderate compression of the transection line. The pancreatic duct should be separately closed during the procedure by a separate suture. Despite all approaches, fistula development after distal pancreatecomy remains an ongoing problem. Fistula rates between 12 and 40% are reported [23-25]. There is no need or evidence for any further covering of the resection margin by sealants or patches [26]. To address this clinical problem, remnant closure by sutures after sharp dissection has currently been compared to stapler dissection in a randomized controlled study (DISPACT trial) in a multicenter approach including 21 European centers and 360 patients [26]. The preliminary results of this study showed no significant differences in both arms with regard to the primary end point fistula after 30 days [27].

### 2.3. Total Pancreatectomy

From the oncological point of view, extensive main-duct IPMNs, IPMNs with progression to carcinoma, familial or multifocal pancreatic cancer as well as multiple metastases in the pancreas are indications for a primary total pancreatectomy. Furthermore, this procedure may be necessary to achieve a tumor-free resection margin and R0 situation in centrally localized tumors of the pancreatic body [28-31]. In the latter case, the resection might be performed as a two-part procedure with an initial head resection followed by the distal resection. In all other cases, removal of the gland as an *en bloc* pancreatectomy should be performed to avoid pancreatic transection with the risk of tumor cell spilling.

## 3. Lymphadenectomy

Lymphadenectomy during PDAC resections is well-defined for partial duodenopancreatectomy, which has been subject of national guidelines and international consensus statements such as the German “S3 guideline”, the Japanese guideline or the guidelines published by the National Comprehensive Cancer Network in the US [32,33]. For distal and total pancreatectomy, there are less consensus based recommendations, however lymphadenectomy is an essential step during these procedures as well.

Different classifications of the peripancreatic lymph nodes have been used by various authors. These classifications range from pure descriptions to approaches that are based on functional considerations [34]. The most commonly accepted system has been established by the Japanese Pancreas Society [35]. According to this system, the peripancreatic lymph nodes can be divided into three groups (1st, 2nd, 3rd order) of regional lymph nodes that are further subdivided in some positions such as the hepato-duodenal ligament (group 12a, b, c, h, p). This classification can be helpful not only clinically to describe lymph node spread in detail, but also to make studies on lymph node dissection comparable.

The clinical and prognostic value of the lymph node status has been investigated in several studies from the histopathological and oncological point of view [36-43]. Two aspects have to be considered that are presently part of a controversial discussion: the overall number of affected lymph nodes as the histopathological correlate of lymph node spread and the lymph node ratio as a calculated parameter that may reflect the relation of spread in a different way. With regard to the above mentioned anatomical system of directly pancreas surrounding lymph nodes and second level nodes, a recent study has investigated if the mechanism of spread has an impact on prognosis [39]. The authors reviewed 517 pancreatic cancer patients with regard to spread into the adjacent or regional nodes. They could not demonstrate a general prognostic difference between metastases that occurred in adjacent or regional nodes. In contrast to this irrelevance in location, the number of positive lymph nodes was shown to have an important impact on long-term outcome. In patients with one positive node, long-term survival was equal to patients with an N0 stage, whereas two or more positive nodes were associated with a significantly poorer survival, regardless to the total number of positive nodes. This observation was similarly made in distal pancreatectomy as mentioned below with a cutoff level of three or more positive nodes. These results underline the importance of lymph node dissection, as in case of only one or two positive nodes the radical removal leads to a dramatically improved prognosis, compared to patients with residual tumor manifestation or merely palliative therapy.

Lymph node ratio is a commonly used term initially introduced to characterize lymphatic tumor load and create a prognostic parameter independent from the rough estimation N0 *vs.* N1 or the overall number of positive lymph nodes [37,38]. A large number of studies have dealt with the topic of the prognostic lymph node ratio cutoff. Usually the number of 0.2 is accepted as the separation level that indicates poor survival [37]. However, recent publications have reached different conclusions regarding the prognostic value of total lymph node count and lymph node ratio. As mentioned in detail above, a situation with one or two positive lymph nodes has been shown to be prognostically equivalent to a N0 stage, no matter which total number of lymph nodes are resected [37]. This observation is not in accordance with the study published by Bhatti *et al.* [38], who found an independent predictive value of the lymph node ratio ≤0.2 for long term survival in their multivariate analysis of 84 patients but could not demonstrate an impact of total lymph node number. Similar results were obtained by Slidell *et al.* [42] in the largest available series that included 4000 patients. They comfirmed the lymph node ratio cutoff of 0.2 as a strong predictive value for survival. The underlying problem which may explain these contrary conclusions may be the variable number of lymph nodes removed during resection. As the number of histopathologically examined lymph nodes in the study by Slidell *et al.* [42] shows a variety between 0 and 30 nodes with a median number of 7, the ratio is strongly dependent on the total number of removed nodes. Therefore, a comparison of the parameters and the different studies has to be seen with regard to this aspect. In general, a minimal required number of 10–12 lymph nodes should be routinely found and prepared in the resection specimen to make a valid statement about the lymph node stage [42,43]. Under the hypothesis of this number, one or two positive nodes would have the same prognostic value as a ratio of 0.1–0.2, making both parameters a similar prognostic value.

The para-aortic lymph nodes (Group 16 according to the Japanese Pancreas Society) represent a group that has to be specifically regarded in terms of prognostic value. Although positive nodes in this group are considered as distant metastases (M1 disease) by many authors, their prognostic impact remains controversial. A review by Glanemann *et al.* [53] showed that patients with tumor spread to these nodes show a significant poorer survival which implies that these patients should not be resected in case of a positive sampling during surgical exploration. However, a study by Shrikande *et al.* [52] compared outcome after resection for M1 pancreatic cancer, including a subgroup with positive para-aortal lymph nodes. In this study, survival of these patients was significantly better and comparable to node-negative patients than in the subgroup with resected liver or peritoneal metastases. Therefore, it seems difficult to draw a final conclusion on this issue.

### 3.1. Lymphadenectomy in Partial Pancreatico-Duodenectomy

Partial duodenopancreatectomy includes a standardized lymphadenectomy, which contains the lymph nodes of the hepato-duodenal ligament (group 12), along the common hepatic artery (group 8), portal vein (group 12) and the cranial portion of superior mesenteric vein (group 4–6) as well as right-sided lymphnodes of the celiac trunk (group 9) and along the right side of the superior mesenteric artery (group 3) [32]. The impact of extended lymph node dissection (*i.e.*, in the interaortocaval space, left-sided of the celiac trunk and superior mesenteric artery) has been well investigated in four randomized controlled trials between 1998 and 2005 [44-47]. Although there were certain differences in the studies with regard to the number of resected lymph nodes (20 *vs.* up to 40), three of the authors could not show any survival difference in the study collectives, neither in N0 nor in N1 patients that underwent standard or extended resections. Only Pedrazzoli *et al.* [44] found a survival benefit of 7 months in the subgroup analysis for N1 patients that underwent extended resection. Furthermore, all authors except for Pedrazzoli *et al.* observed a significantly increased surgical morbidity or decreased quality of life in the postoperative follow-up.

A metaanalysis published in 2007 [48] analyzed these studies, including an overall number of 297 *vs.* 311 patients, with regard to their individual scientific quality and results. No benefit for an extended approach of lymph node dissection could be concluded in terms of tumor control and survival. Furthermore, an increased rate of perioperative complications and a decreased quality of life was demonstrated. Therefore, with regard to these studies and consequently based on a level 1 evidence, the concept of ultra-radical lymphadenectomy should be abandoned and defined standardized lymphnode dissection should be performed during partial duodenopancreatectomy.

### 3.2. Lymphadenectomy in Distal Pancreatectomy

Lymph node involvement in tumors located in the body or tail of the gland is most frequently observed in the lymph nodes attached to the pancreas in the resected specimen [41]. Further frequent sites are the nodes along the splenic artery, the paraaortic area and along the inferior margin of the pancreas as well as along the superior mesenteric artery.

The extent of lymph node dissection in distal pancreatectomy has been defined in 1999 by a European consensus statement [22] using the Japanese Pancreas Society classification [35]. The standard lymphadenectomy includes regional lymph nodes attached to the pancreas along the inferior margin, the celiac trunk and the splenic artery as well as the hilum of the spleen [22,49-51]. “Radical” lymphadenectomy is defined as an additional dissection of the lymphatic tissue along the hepatic artery and the interaorto-caval space including Gerota's fascia [22]. This extended resection is not a standard procedure but may be suitable as an individual approach in situations when these lymph nodes are macroscopically suspicious during exploration [52], although metastases in the aorto-caval space are considered as distant metastases by many authors due to the often poor prognosis [53,54].

A recent study has investigated the prognostic impact of lymph node metastases in the regional lymph nodes [41]. The authors showed that positive lymph node status was an independent factor predicting survival after distal pancreatectomy. Interestingly, they could not only demonstrate the prognostic value of positive lymph nodes themselves. When the lymph node positive patients were subdivided into groups with one or two involved nodes attached to the pancreas and three or more nodes, survival of the latter group was significantly worse. One or two positive nodes were not associated with an impairment of the prognosis but had an equal survival to those patients with an N0 situation. This observation shows that a routine removal of the directly attached and peripancreatic nodes is a highly important part of the resection to achieve best oncological outcome. In contrast, a more extended lymphadenectomy cannot be recommended from the available data, as this is, comparable to the setting of pancreatic head resection, associated with an increase in morbidity without proven oncological benefit.

### 3.3. Lymphadenectomy in Total Pancreatectomy

Lymphadenectomy during total pancreatectomy is, comparable to distal pancreatectomy, not fully standardized [55,56]. However, it seems reasonable to regard total pancreatectomy as a combination of partial duodenopancreatectomy and distal resection and therefore combine the approaches of lymph node dissection of both procedures. This includes the dissection of the hepatoduodenal ligament, along the hepatic artery, both sides of the celiac trunk, the splenic artery as well as the inferior pancreatic margin and will usually result in 30–50 lymphnodes included in the resected specimen. Interaortocaval lymph node resection during total pancreatectomy is an extended approach and must not be performed as a routine procedure, as morbidity may be increased especially in terms of lymphatic fistula development and therapeutically challenging diarrhea and intestinal discomfort which can severely impair patients' recovery and postpone or even inhibit the start of an adjuvant chemotherapy. As the prognostic oncological value of ultra-radical dissection remains questionable, this approach cannot be recommended during total pancreatectomy.

## 4. Vascular Resections

**Figure 2.**
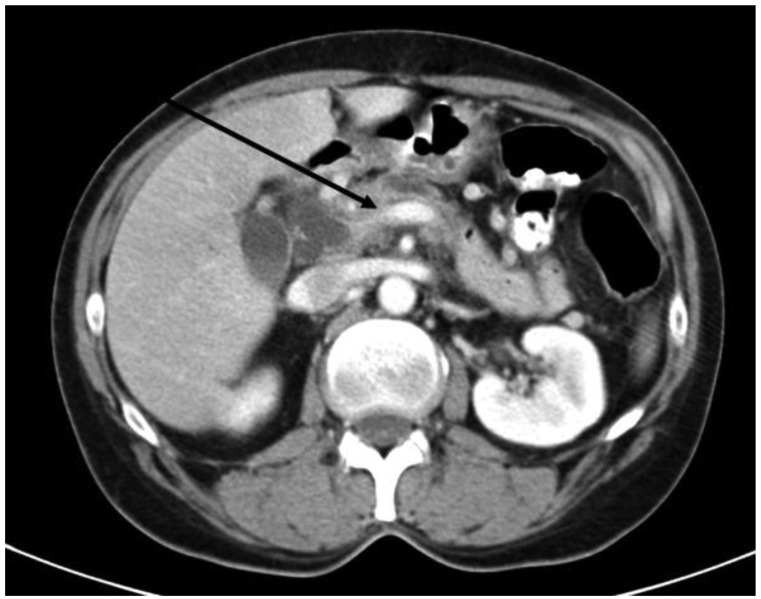
CT scan showing pancreatic head cancer with infiltration of the portal vein confluens (black arrow). Technically resectable finding (partial duodeno-pancreatectomy with segmental portal vein resection).

Depending on the localization and growth pattern of the tumor, two types of adherence or infiltration of vascular structures need to be regarded: Venous vessel involvement of the superior mesenteric and/or portal vein and involvement of the celiac trunk or the superior mesenteric artery as the major upper GI arterial vessels (Figure 2).

### 4.1. Venous Resections

From the initially anecdotal description in the 1970s, resection of the portal vein or the superior mesenteric vein have gained wide acceptance in centers around the world. Depending on the length of tumor adherence, the portal and superior mesenteric vein can be reconstructed by a direct anastomosis or the interposition of a graft. Both procedures can be performed safely, which has been demonstrated in large series that showed surgical morbidity and mortality rates comparable to pancreatic head resections without vascular involvement [57-59]. In a systematic review published in 2006 [60], 52 manuscripts with more than 6300 patients were included in whom PDAC resection was performed. This collective included 1646 patients (26%) who underwent synchronous portal—superior mesenteric vein resection mainly together with partial pancreaticoduodenectomy (71%) or total pancreatectomy (24%). Median operation time was approximately 8.5 hours with a median blood loss of 1750 mL, perioperative mortality of 5.9% and overall morbidity of 42 % (9% to 78%).

Technically, venous resection can be performed as a tangential resection of the portal vein if this is possible. Mostly, tumor infiltration reaches the vein from the right circumference, sometimes giving the surgeon the opportunity to resect a small patch and close the defect directly without a hemodynamically relevant stenosis (Figure 2). However, the congestion of the venous drainage of the small bowel needs to be ensured which is certainly possible by direct flow measurement on the one hand and the macroscopic judgement of the experienced surgeon during the remaining operation time, which is usually about two hours it takes to complete reconstruction of the pancreatic, bile duct and duodenal or stomach anastomosis. If the tangential resection is not possible, the mesenteric root can be mobilized completely by resolving the attachment of the right hemicolon to the retroperitoneal adhesions [57]. A segmental portal vein resection can be performed and continuity of the vessel can be restored by a direct end-to-end anastomosis. When the resected length cannot be bridged by the direct anastomosis, a vascular graft needs to be inserted. In recent clinical studies, no difference in surgical outcome and long-term survival was shown when different types of venous reconstruction (venorrhaphy, end-to-end anastomosis, graft insertion) were compared [58].

A tumor-related complete obstruction of the portal vein must not be regarded as a general obstacle for a resection. Although surgical preparation may be more difficult due to the collateral vessels, the restoration of the portal venous flow after resection and anastomosis offers an adequate drainage of the bowel despite the removal of most of the collateral vessels that may be necessary during the preparation. Oncological outcome in patients with venous resections has been shown to be similar without increased rates of local or systemic failure [60]. Overall, histological tumor invasion of the portal vein is evident in approximately two out of three resected specimens, varying between 3 and 86% in the different series. The reported long-term survival in the review by Siriwardana *et al.* [60] of 1,351 patients after portal—superior mesenteric vein resection was 13 months, with 1-, 3-, and 5- year overall survival rates of 50%, 18%, and 8%. This demonstrates that resection of the portal or superior mesenteric vein is potentially curative and the involvement of the mesenteric or portal vein seems to be rather a consequence of the tumor located close to these structures than a reflection of an uncommonly aggressive tumor biology. Since perioperative morbidity and mortality rates are favourable and long-term survival after these resections is much better compared to patients with only palliative management, resection of the portal and superior mesenteric vein can be regarded as a standard procedure in experienced hands and should be performed in a routine setting to achieve a complete removal of the tumor. This has meanwhile been generally accepted and is explicitly stated in national guidelines such as the German consensus publication of 2007 [32], which underlines the importance and binding character of this approach that can also be recommended during multivisceral procedures [61].

### 4.2. Arterial Resections

**Figure 3.**
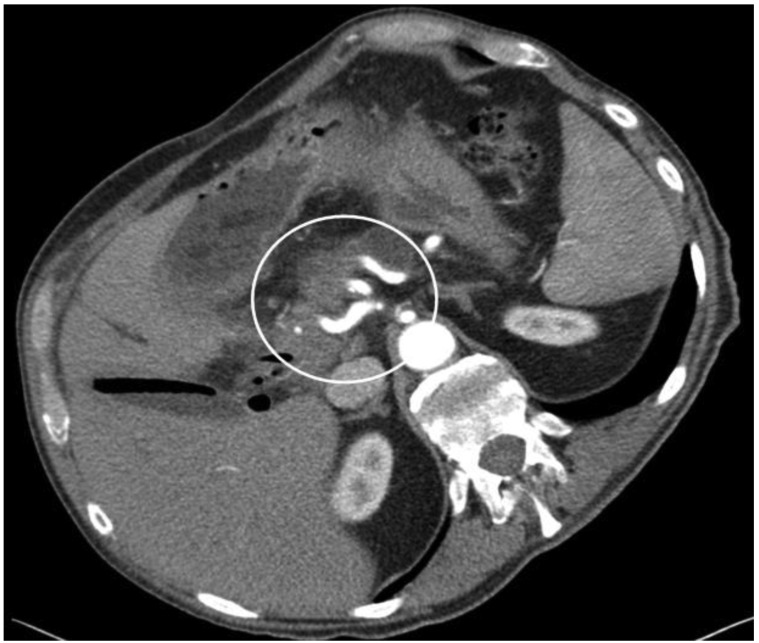
CT scan showing pancreatic head cancer with infiltration of the celiac trunk. Infiltration of common hepatic, left gastric and splenic artery (white circle, left, middle and right vessel). Technically not resectable finding.

In contrast to venous tumor adhesion, arterial infiltration of the celiac axis (Figure 3) or the superior mesenteric artery must be regarded as a symptom of biologically aggressive tumor spread. Therefore, decision to perform a surgical resection in this situation is a highly individual decision. The resection of the celiac axis or the superior mesenteric artery has been performed since the 1970s in selected patients but is still regarded as an extraordinary approach in PDAC surgery [62-65].

If the superior mesenteric artery is involved in the tumor process, this is rather a general exclusion criterion for resection and has only been reported in few patients. In contrast, tumor adherence or infiltration along the celiac axis must not be considered as generally irresectable [66]. To evaluate arterial infiltration along the superior mesenteric artery, the “artery-first” approach can be useful [67], which describes the preparation of the superior mesenteric artery as the initial step before further preparation and mobilization of the pancreatic head or tail to rule out any tumor infiltration.

However, arterial resections have to be evaluated differently in pancreatico-duodenectomy or distal pancreatectomy. This also implies the differentiation of resection without re-vascularization and resection with direct anastomosis or graft insertion to replace the resected vessel. In a recent review, the role of arterial resection has been critically evaluated including all currently available studies [68].

In pancreatico-duodenectomy with resection of the superior mesenteric artery, only five studies were identified including a total number of less than 30 patients. All authors showed that the resection is technically possible, grafting with the saphenous vein was the most commonly used method for reconstruction. However, morbidity of this approach is high and the oncological outcome is not yet convincing from the limited evidence. Celiac axis or hepatic artery resection in pancreatico-duodenectomy is performed more often. The available literature on this topic includes approximately 200 patients [53]. Surgical morbidity is up to 40%, mortality in pancreatico-duodenectomy with arterial resection ranges from 0–35%, showing the inconsistent data basis of this approach. Long-term survival in these studies shows three-year survival rates of 8–35%. Five-year survival was 0% in most publications, only one study reported a 20% survival [63]. These results show that arterial invasion of pancreatic cancer located in the pancreatic head has to be regarded as a rather poor prognostic factor.

In distal pancreatectomy, case series regarding celiac axis resection included 1–13 patients with a median survival of 9–14 months [69, 70]. In a recent publication [69], long term survival after this approach was compared to standard distal pancreatectomy and was equal in both procedures, which underlines that arterial resection in distal pancreatectomy seems to be feasible and reasonable. From the technical point of view, the celiac trunk might be resected down to its aortal orifice [69]. As long as the proper hepatic artery can be preserved, a reconstruction is possible. This can be done with an interposition of any arterial vessel of the celiac axis or a venous interposition graft. Arterial liver perfusion should be controlled by regular duplex examinations and restored aggressively in case of a vessel occlusion. Arterial hepatic perfusion failure may otherwise cause acute problems postoperatively in terms of liver ischemia, necrosis and infection and is a risk factor for bile-duct associated complications in the long-term follow up [71]. In summary, arterial resection can be carried out safely in experienced hands but is not based on high-quality scientific data so far.

## 5. Multivisceral Resections

About 35% of all potentially resectable PDAC patients present with locally advanced tumors with involvement of surrounding structures and organs. The benefit of radical surgical resection in this situation has been addressed in several publications [61,72]. Older reports showed that surgical morbidity in extended resection was increased and survival benefit limited, making this approach questionable. In contrast, more recent publications [61] demonstrate that en-bloc resection of contiguously involved organs can be performed safely. There is no difference regarding perioperative morbidity (35%) and mortality (3%) compared to standard resection. While operation time is clearly longer due to the extended resection including mesocolon, colon, adrenal glands, liver, and stomach, blood loss and hospital stay are not different from what is observed after the standard procedure. Major aim of any extended resection must be the achievement of a curative situation. With a five-year survival rate of 16% and a median survival of 26 months, results of the multivisceral approach are comparable to long-term survival rates of patients with standard resection and, above all, much better than the median survival of 6–9 months reported for patients who are not resected at all [1].

## 6. Neoadjuvant Therapy

Neoadjuvant therapy in pancreatic cancer is currently under discussion with regard to the differing clinical situation. On one hand a not resectable or borderline resectable locally advanced tumor represents an indication for preoperative treatment, on the other hand, pretreatment can be performed in limited tumors which can be radically resected to improve local control and reduce the risk of recurrence.

In case of locally advanced tumors with adherence to the celiac trunk or the superior mesenteric artery, a neoadjuvant treatment can be performed following different study protocols and is not standardized yet. In many protocols, gemcitabine is combined with a 50–60 Gy radiation over a 6-week period, followed by 4–6 weeks interval to await downsizing and development of fibrosis as a consequence of the therapy. After re-staging, patients should be subjected to surgical exploration as long as no signs of systemic tumor spread are visible. Using this approach, in 33–50% of all primarily irresectable patients, a radical resection is possible which achieves R0 resection rates comparable to standard resections [66,73-75].

In case of resectable carcinomas, there is no evidence for neoadjuvant treatment to date. A recent review summarized the published data on this topic including 35 studies with a median number of 32 patients per study [76]. Although there is a wide variety of neoadjuvant therapies with different chemotherapeutic agents and radiotherapy, no advantage in terms of tumor response and survival could be shown for the neoadjuvant treatment group in respectable carcinomas. Overall median survival after neoadjuvant therapy followed by resection was 23.3 months compared to 20.1–23.6 months when a primary resection was performed. These findings do not suggest the use of neoadjuvant treatment in case of respectable pancreatic cancer. However, the methodological problems implied in the current literature needs to be taken into account. Especially different definitions of resectability and non-standardized treatment protocols make a comparison between the studies difficult.

## 7. Recurrence Resections

Localized recurrence in pancreatic cancer may be an indication for resection in selected patients. As conventional cross-sectional imaging by CT scan is often unspecific with regard to postoperative follow-up in resected patients, early detection of local tumor recurrence remains a diagnostic challenge. Two recent studies have demonstrated that fluorodeoxyglucose-positron emission tomography (FDG-PET) is a useful tool when addressing this problem [77,78]. Its sensitivity and specificity seems to be significantly higher in early detection of tumor relapse, which may lead to a change in treatment strategy and surgical reexploration in selected patients. Although a large number of tumor recurrences is located close to the arterial vessels and therefore not resectable, recent studies support the concept of surgical exploration and resection whenever possible [79-81]. This approach can be combined with intraoperative radiotherapy of the tumor bed to reduce the risk of another recurrence at the resection site. In case of local irresectability, intraoperative radiation can be performed with a palliative intention in terms of tumor reduction and pain control. An extended resection of the recurrent tumor with arterial vessels does not seem to be justified as the chance for a radical tumor removal is poor. The available studies report successful resection rates of 50% with acceptable surgical morbidity and suggest a survival benefit for those patients, especially in situations with a long time interval (>9–12 months) between the initial tumor diagnosis and the recurrence manifestation [82]. As these are observational studies, there is no proven evidence for this approach today and larger controlled trials are required to evaluate long-term oncological value.

## 8. Laparoscopic Resections in Pancreatic Cancer

The role of laparoscopic resections in pancreatic cancer is controversially discussed. While distal laparoscopic pancreatectomy gains growing acceptance worldwide, only few reports have been published on partial pancreatico-duodenectomy [82-90].

The laparoscopic approach for distal pancreatectomy is usually performed using 4–5 trocars and stapler dissection of the pancreas and shows similar operative morbidity rates and outcome compared to the open approach in experienced hand [25]. The possible laparoscopic advantages, especially faster recovery of the patients, less pain medication and better cosmetic results, are currently evaluated in larger series. So far, no randomized trial has been performed. Furthermore, there are no larger series reporting on the oncological issues in terms of radical resection, number of removed lymph nodes and long-term outcomes of this procedure. The three largest studies published in recent years included overall collectives of 103–159 patients undergoing laparoscopic distal pancreatectomy. Among these, only 4, 13 and 16 pancreatic cancers were found [25,89,90]. This limited data does not allow conclusions to be drawn on the oncological feasibility of laparoscopic distal pancreatectomy.

Partial pancreatico-duodenectomy has been performed laparoscopically in only few institutions around the world with very small numbers of patients and cannot be recommended as a standard approach in pancreatic cancer [82].

## 9. Quality of Life (QoL)

Long-term quality of life (QoL) after pancreatic cancer resection must be seen as a combination of surgical and oncological issues. From the surgical point of view, the patient's postoperative gastrointestinal function and nutritional status depends on gastric emptying function, which can be impaired in the early course by delayed gastric emptying, as well as malabsorption, usually caused by exocrine insufficiency [91]. Furthermore, postoperative diabetes mellitus may require oral antidiabetic and/ or insulin therapy [92,93]. In addition, QoL can be impaired by side effects of adjuvant therapies, including general weakness, nausea, vomiting and diarrhea [92]. In general, the surgical QoL returns to the preoperative level within six months postoperatively. There are several randomized controlled studies in which QoL following pylorus-preserving and classical partial pancreatico-duodenectmy have been investigated [12,94-96]. Regarding these studies, the pylorus-preserving procedure seems to have a better outcome in terms of nausea, vomiting and postoperative weight gain as well as working ability after six months [12]. However, overall QoL was not significantly different and study results showed a wide heterogeneity. A valid conclusion is difficult due to different questionnaires and points of time used in the studies. Due to perioperative advantages (operation time, blood loss) of the pylorus preserving procedure, this seems to be favorable as long-term results are at least comparable to the classical resection and could be superior in long-term outcome as well.

After total pancreatectomy, complete endo- and exocrine insufficiency are the two major factors that affect long-term QoL. Both can successfully be treated by supplementation of oral enzyme application and insulin, respectively. In a study collective of 147 patients that underwent total pancreatectomy for mainly oncological indications, QoL was comparable to patients undergoing a Whipple procedure with a median 23 months follow-up period [31]. Another study included 34 long-time survivors after total pancreatectomy with a mean follow-up of 7.5 years [93]. This study demonstrated that diabetes control is a major topic for these patients and requires recurrent hospitalizations. Other QoL issues were not significantly affected. Both studies show that total pancreatectomy must not be associated with any severe deficiencies in routine activities and underline the importance of well-educated endocrine and exocrine substitution therapy to achieve a long-lasting good QoL.

## 10. Conclusion

In conclusion, pancreatic resections for cancer can be performed with considerable safety and low pancreas-specific complication rates. Venous resection should be performed when there are no other contraindications, multivisceral and arterial resections might be justified in selected cases as well as resection of tumor recurrences, whereas extended lymphadenectomy has not proven useful in pancreatic adenocarcinoma. Additionally, all surgical approaches should be part of interdisciplinary multimodal treatment concepts to improve patient prognosis.

## Figures and Tables

**Table 1.** Currently available randomized controlled trials on pylorus-preserving *vs.* classical Whipple resection.

**Author [Ref.]**	**Year**	**RCT**	**N**	**DGE**	**QoL**
Paquet [97]	1998	+	pp 17	35.3%	NR
			cl 23	4.3%	
Wenger [95]	1999	+	pp 24	NR	pp > cl
			cl 24		(in several items)
Bloechle [96]	1999	+	pp 23	34.8%	pp > cl
			cl 21	9.5%	(in several items)
Tran [98]	2004	+	pp 85	22.4%	NR
			cl 80	22.5%	
Lin [99]	2005	+	pp 14	42.9%	NR
			cl 19	0%	
Seiler [97]	2005	+	pp 64	31.1%	n.s.
			cl 67	45.5%	

## References

[1] Hariharan D., Saied A., Kocher H.M. (2008). Analysis of mortality rates for pancreatic cancer across the world. HPB (Oxford).

[2] Jemal A., Siegel R., Xu J. (2010). Cancer statistics, 2010. CA Cancer J. Clin..

[3] Gaedcke J., Gunawan B., Grade M., Szöke R., Liersch T., Becker H., Ghadimi B.M. (2010). The mesopancreas is the primary site for R1 resection in pancreatic head cancer: Relevance for clinical trials. Langenbecks Arch. Surg..

[4] Neoptolemos J.P., Stocken D.D., Friess H., Bassi C., Dunn J.A., Hickey H., Beger H., Fernandez-Cruz L., Dervenis C., Lacaine F., Falconi M., Pederzoli P., Pap A., Spooner D., Kerr D.J., Büchler M.W. (2004). European Study Group for Pancreatic Cancer. A randomized trial of chemoradiotherapy and chemotherapy after resection of pancreatic cancer. N. Engl. J. Med..

[5] Oettle H., Post S., Neuhaus P., Gellert K., Langrehr J., Ridwelski K., Schramm H., Fahlke J., Zuelke C., Burkart C., Gutberlet K., Kettner E., Schmalenberg H., Weigang-Koehler K., Bechstein W.O., Niedergethmann M., Schmidt-Wolf I., Roll L., Doerken B., Riess H. (2007). Adjuvant chemotherapy with gemcitabine vs observation in patients undergoing curative-intent resection of pancreatic cancer: randomized controlled trial. JAMA.

[6] Renouf D., Moore M. (2010). Evolution of systemic therapy for advanced pancreatic cancer. Expert. Rev. Anticancer Ther..

[7] Smeenk H.G., Tran T.C., Erdmann J., van Eijck C.H., Jeekel J. (2005). Survival after surgical management of pancreatic adenocarcinoma: does curative and radical surgery truly exist?. Langenbecks Arch. Surg..

[8] Büchler M.W., Wagner M., Schmied B.M., Uhl W., Friess H., Z'graggen K. (2003). Changes in morbidity after pancreatic resection: toward the end of completion pancreatectomy. Arch. Surg..

[9] Birkmeyer J.D., Siewers A.E., Finlayson E.V., Goodney P.P., Wennberg D.E., Lucas F.L. (2002). Hospital volume and surgical mortality in the United States. N. Engl. J. Med..

[10] McPhee J.T., Hill J.S., Whalen G.F., Zayaruzny M., Litwin D.E., Sullivan M.E., Anderson F.A., Tseng J.F. (2007). Perioperative mortality for pancreatectomy: a national perspective. Ann. Surg..

[11] Diener M.K., Knaebel H.P., Heukaufer C., Antes G., Buchler M.W., Seiler C.M. (2007). A systematic review and meta-analysis of pylorus-preserving versus classical pancreaticoduodenectomy for surgical treatment of periampullary and pancreatic carcinoma. Ann. Surg..

[12] Diener M.K., Heukaufer C., Schwarzer G., Seiler C.M., Antes G., Buchler M., Knaebel H.P. (2008). Pancreaticoduodenectomy (classic Whipple) versus pylorus-preserving pancreaticoduo-denectomy (pp Whipple) for surgical treatment of periampullary and pancreatic carcinoma. Cochrane Database Sys.t Rev..

[13] Bassi C., Dervenis C., Butturini G., Fingerhut A., Yeo C., Izbicki J., Neoptolemos J., Sarr M., Traverso W., Buchler M.W. (2005). International Study Group on Pancreatic Fistula Definition. Postoperative pancreatic fistula: an international study group (ISGPF) definition. Surgery.

[14] Reid-Lombardo K.M., Farnell M.B., Crippa S., Barnett M., Maupin G., Bassi C., Traverso L.W. (2007). Pancreatic Anastomotic Leak Study Group. Pancreatic anastomotic leakage after pancreaticoduodenectomy in 1,507 patients: a report from the Pancreatic Anastomotic Leak Study Group. J. Gastrointest. Surg..

[15] Daskalaki D., Butturini G., Molinari E., Crippa S., Pederzoli P., Bassi C. (2011). A grading system can predict clinical and economic outcomes of pancreatic fistula after pancreaticoduodenectomy: results in 755 consecutive patients. Langenbecks Arch. Surg..

[16] Peng S., Wang J., Li J., Mou Y., Liu Y., Cai X. (2004). Binding pancreaticojejunostomy—A safe and reliable anastomosis procedure. HPB (Oxford)..

[17] Poon R.T., Fan S.T., Lo C.M., Ng K.K., Yuen W.K., Yeung C., Wong J. (2007). External drainage of pancreatic duct with a stent to reduce leakage rate of pancreaticojejunostomy after pancreaticoduodenectomy: a prospective randomized trial. Ann. Surg..

[18] Wente M.N., Shrikhande S.V., Kleeff J., Müller M.W., Gutt C., Büchler M.W., Friess H. (2006). Management of early hemorrhage from pancreatic anastomoses after pancreatico-duodenectomy. Dig. Surg..

[19] Hartel M., Wente M.N., Hinz U., Kleeff J., Wagner M., Müller M.W., Friess H., Büchler M.W. (2005). Effect of antecolic reconstruction on delayed gastric emptying after the pylorus-preserving Whipple procedure. Arch. Surg..

[20] Tani M., Terasawa H., Kawai M., Ina S., Hirono S., Uchiyama K., Yamaue H. (2006). Improvement of delayed gastric emptying in pylorus-preserving pancreaticoduodenectomy: results of a prospective, randomized, controlled trial. Ann. Surg..

[21] Strasberg S.M., Drebin J.A., Linehan D. (2003). Radical antegrade modular pancreatosplenectomy. Surgery..

[22] Pedrazzoli S., Beger H.G., Obertop H., Andrén-Sandberg A., Fernández-Cruz L., Henne-Bruns D., Lüttges J., Neoptolemos J.P. (1999). A surgical and pathological based classification of resective treatment of pancreatic cancer. Summary of an international workshop on surgical procedures in pancreatic cancer. Dig. Surg..

[23] Knaebel H.P., Diener M.K., Wente M.N., Büchler M.W., Seiler C.M. (2005). Systematic review and meta-analysis of technique for closure of the pancreatic remnant after distal pancreatectomy. Br. J. Surg..

[24] Andrén-Sandberg A., Wagner M., Tihanyi T., Löfgren P., Friess H. (1999). Technical aspects of left-sided pancreatic resection for cancer. Dig. Surg..

[25] Fernández-Cruz L., Orduña D., Cesar-Borges G., Angel López-Boado M. (2005). Distal pancreatectomy: en-bloc splenectomy vs spleen-preserving pancreatectomy. HPB (Oxford).

[26] Diener M.K., Knaebel H.P., Witte. S.T., Rossion I., Kieser M., Buchler M.W., Seiler C.M. (2008). DISPACT Trial Group. DISPACT trial: a randomized controlled trial to compare two different surgical techniques of DIStal PAnCreaTectomy - study rationale and design. Clin. Trials.

[27] Friess H. (2010). Distal Pancreatectomy—Stapler Versus Suture (DISPACT).

[28] Keck T., Hopt UT. (2008). Total pancreatectomy: renaissance of a surgical procedure. Chirurg.

[29] Sohn T.A., Yeo C.J., Cameron J.L., Hruban R.H., Fukushima N., Campbell K.A., Lillemoe K.D. (2004). Intraductal papillary mucinous neoplasms of the pancreas: An updated experience. Ann. Surg..

[30] Inagaki M., Obara M., Kino S., Goto J., Suzuki S., Ishizaki A., Tanno S., Kohgo Y., Tokusashi Y., Miyokawa N., Kasai S. (2007). Pylorus-preserving total pancreatectomy for an intraductal papillary-mucinous neoplasm of the pancreas. J. Hepatobiliary Pancreat. Surg..

[31] Müller M.W., Friess H., Kleeff J., Dahmen R., Wagner M., Hinz U., Breisch-Girbig D., Ceyhan G.O., Büchler M.W. (2007). Is there still a role for total pancreatectomy?. Ann. Surg..

[32] Adler G., Seufferlein T., Bischoff S.C., Brambs H.J., Feuerbach S., Grabenbauer G., Hahn S., Heinemann V., Hohenberger W., Langrehr J.M., Lutz M.P., Micke O., Neuhaus H., Neuhaus P., Oettle H., Schlag P.M., Schmid R., Schmiegel W., Schlottmann K., Werner J., Wiedenmann B., Kopp I. (2007). S3-Guidelines “Exocrine pancreatic cancer” 2007. Z. Gastroenterol..

[33] Bilimoria K.Y., Bentrem D.J., Lillemoe K.D., Talamonti M.S., Ko C.Y. (2009). Pancreatic Cancer Quality Indicator Development Expert Panel, American College of Surgeons. Assessment of pancreatic cancer care in the United States based on formally developed quality indicators. J. Natl. Cancer Inst..

[34] O'Morchoe C.C. (1997). Lymphatic system of the pancreas. Microsc. Res. Tech..

[35] Japan Pancreas Society (2003). Classification of Pancreatic Carcinoma.

[36] Birk D., Beger H.G. (1999). Lymph-node dissection in pancreatic cancer-what are the facts?. Langenbecks Arch. Surg..

[37] Murakami Y., Uemura K., Sudo T., Hayashidani Y., Hashimoto Y., Nakashima A., Yuasa Y., Kondo N., Ohge H., Sueda T. (2010). Number of metastatic lymph nodes, but not lymph node ratio, is an independent prognostic factor after resection of pancreatic carcinoma. J. Am. Coll. Surg..

[38] Bhatti I., Peacock O., Awan A.K., Semeraro D., Larvin M., Hall R.I. (2010). Lymph node ratio versus number of affected lymph nodes as predictors of survival for resected pancreatic adenocarcinoma. World J. Surg..

[39] Konstantinidis I.T., Deshpande V., Zheng H., Wargo J.A., Fernandez-del Castillo C., Thayer S.P., Androutsopoulos V., Lauwers G.Y., Warshaw A.L., Ferrone C.R. (2010). Does the mechanism of lymph node invasion affect survival in patients with pancreatic ductal adenocarcinoma?. J. Gastrointest. Surg..

[40] Massucco P., Ribero D., Sgotto E., Mellano A., Muratore A., Capussotti L. (2009). Prognostic significance of lymph node metastases in pancreatic head cancer treated with extended lymphadenectomy: not just a matter of numbers. Ann. Surg. Oncol..

[41] Fujita T., Nakagohri T., Gotohda N., Takahashi S., Konishi M., Kojima M., Kinoshita T. (2010). Evaluation of the prognostic factors and significance of lymph node status in invasive ductal carcinoma of the body or tail of the pancreas. Pancreas.

[42] Slidell M.B., Chang D.C., Cameron J.L., Wolfgang C., Herman J.M., Schulick R.D., Choti M.A., Pawlik TM. (2008). Impact of total lymph node count and lymph node ratio on staging and survival after pancreatectomy for pancreatic adenocarcinoma: A large, population-based analysis. Ann. Surg. Oncol..

[43] Pawlik T.M., Gleisner A.L., Cameron J.L., Winter J.M., Assumpcao L., Lillemoe K.D., Wolfgang C., Hruban R.H., Schulick R.D., Yeo C.J., Choti M.A. (2007). Prognostic relevance of lymph node ratio following pancreaticoduodenectomy for pancreatic cancer. Surgery.

[44] Pedrazzoli S., DiCarlo V., Dionigi R., Mosca F., Pederzoli P., Pasquali C., Klöppel G., Dhaene K., Michelassi F. (1998). Standard versus extended lymphadenectomy associated with pancreatoduodenectomy in the surgical treatment of adenocarcinoma of the head of the pancreas: A multicenter, prospective, randomized study. Ann. Surg..

[45] Yeo C.J., Cameron J.L., Sohn T.A., Coleman J., Sauter P.K., Hruban R.H., Pitt H.A., Lillemoe K.D. (1999). Pancreaticoduodenectomy with or without extended retroperitoneal lymphadenectomy for periampullary adenocarcinoma: comparison of morbidity and mortality and short-term outcome. Ann. Surg..

[46] Farnell M.B., Pearson R.K., Sarr M.G., DiMagno E.P., Burgart L.J., Dahl T.R., Foster N., Sargent D.J. (2005). Pancreas Cancer Working Group. A prospective randomized trial comparing standard pancreatoduodenectomy with pancreatoduodenectomy with extended lymphadenectomy in resectable pancreatic head adenocarcinoma. Surgery.

[47] Nimura Y., Nagino M., Kato H., Miyagawa S., Yamaguchi A., Kinoshita T. (2004). Regional versus extended lymph node dissection in radical pancreaticoduodenectomy for pancreatic cancer: A multicenter, randomized controlled trial. HPB.

[48] Michalski C.W., Kleeff J., Wente M.N., Diener M.K., Büchler M.W., Friess H. (2007). Systematic review and meta-analysis of standard and extended lymphadenectomy in pancreaticoduodenectomy for pancreatic cancer. Br. J. Surg..

[49] Brennan M.F., Moccia R.D., Klimstra D. (1996). Management of adenocarcinoma of the body and tail of the pancreas. Ann. Surg..

[50] Shoup M., Conlon K., Klimstra D., Brennan M.F. (2003). Is extended resection for adenocarcinoma of the body or tail of the pancreas justified?. J. Gastrointest. Surg..

[51] Lin C.C., Chen C.L., Cheng Y.F. (2005). Modified extended distal pancreatectomy for carcinoma of body and tail of pancreas. Hepatogastroenterology.

[52] Shrikhande S.V., Kleeff J., Reiser C., Weitz J., Hinz U., Esposito I., Schmidt J., Friess H., Büchler M.W. (2007). Pancreatic resection for M1 pancreatic ductal adenocarcinoma. Ann. Surg. Oncol..

[53] Glanemann M., Shi B., Liang F., Sun X., Bahra M., Jacob D., Neumann U., Neuhaus P. (2008). Surgical strategies for treatment of malignant pancreatic tumors: extended, standard or local surgery?. World J. Surg. Oncol..

[54] Doi R., Kami K., Ito D., Fujimoto K., Kawaguchi Y., Wada M., Kogire M., Hosotani R., Imamura M., Uemoto S. (2007). Prognostic implication of para-aortic lymph node metastasis in resectable pancreatic cancer. World J. Surg..

[55] Ihse I., Anderson H., Andren S. (1996). Total pancreatectomy for cancer of the pancreas: Is it appropriate?. World J. Surg..

[56] Schmidt C.M., Glant J., Winter J.M., Kennard J., Dixon J., Zhao Q., Howard T.J., Madura J.A., Nakeeb A., Pitt H.A., Cameron J.L., Yeo C.J., Lillemoe K.D. (2007). Total pancreatectomy (R0 resection) improves survival over subtotal pancreatectomy in isolated neck margin positive pancreatic adenocarcinoma. Surgery.

[57] Weitz J., Kienle P., Schmidt J., Friess H., Büchler M.W. (2007). Portal vein resection for advanced pancreatic head cancer. J. Am. Coll. Surg..

[58] Müller S.A., Hartel M., Mehrabi A., Welsch T., Martin D.J., Hinz U., Schmied B.M., Büchler M.W. (2009). Vascular resection in pancreatic cancer surgery: survival determinants. J. Gastrointest. Surg..

[59] Martin R.C., Scoggins C.R., Egnatashvili V., Staley C.A., McMasters K.M., Kooby DA. (2009). Arterial and venous resection for pancreatic adenocarcinoma: operative and long-term outcomes. Arch. Surg..

[60] Siriwardana H., Siriwardena A. (2006). Systematic review of outcome of synchronous portal-superior mesenteric vein resection during pancreatectomy for cancer. Br. J. Surg..

[61] Hartwig W., Hackert T., Hinz U., Hassenpflug M., Strobel O., Büchler M.W., Werner J. (2009). Multivisceral resection for pancreatic malignancies: risk-analysis and long-term outcome. Ann. Surg..

[62] Settmacher U., Langrehr J., Husmann I., Eisele R., Bahra M., Heise M., Neuhaus P. (2004). Reconstruction of visceral arteries with homografts in excision of the pancreas. Chirurg.

[63] Yekebas E.F., Bogoevski D., Cataldegirmen G., Kunze C., Marx A., Vashist Y.K., Schurr P.G., Liebl L., Thieltges S., Gawad K.A., Schneider C., Izbicki J.R. (2008). En bloc vascular resection for locally advanced pancreatic malignancies infiltrating major blood vessels: perioperative outcome and long-term survival in 136 patients. Ann. Surg..

[64] Li B., Chen F., Ge X. (2004). Pancreaticoduodenectomy with vascular reconstruction in treating carcinoma of the pancreatic head. Hepatobiliary Pancreat. Dis. Int..

[65] Amano H., Miura F., Toyota N. (2009). Is pancreatectomy with arterial reconstruction a safe and useful procedure for locally advanced pancreatic cancer?. J. Hepatobiliary Pancreat. Surg..

[66] Kleeff J., Friess H., Büchler M.W. (2007). Neoadjuvant therapy for pancreatic cancer. Br. J. Surg..

[67] Weitz J., Rahbari N., Koch M., Buchler M.W. (2010). The “Artery First” Approach for Resection of Pancreatic Head Cancer. J. Am. Coll. Surg..

[68] Chua T.C., Saxena A. (2010). Extended pancreaticoduodenectomy with vascular resection for pancreatic cancer: a systematic review. J. Gastrointest. Surg..

[69] Wu X., Tao R., Lei R., Han B., Cheng D., Shen B., Peng C. (2010). Distal pancreatectomy combined with celiac axis resection in treatment of carcinoma of the body/tail of the pancreas: a single-center experience. Ann. Surg. Oncol..

[70] Wu Y.L., Yan H.C., Chen L.R., Gao S.L., Chen J., Dong X. (2007). Extended Appleby's operation for pancreatic cancer involving celiac axis. J. Surg. Oncol..

[71] Gaujoux S., Sauvanet A., Vullierme M.P., Cortes A., Dokmak S., Sibert A., Vilgrain V., Belghiti J. (2009). Ischemic complications after pancreaticoduodenectomy: Incidence, prevention, and management. Ann. Surg..

[72] Sasson A., Hoffmann J., Ross E., Kagan S.A., Pingpank J.F., Eisenberg B.L. (2002). En bloc resection for locally advanced cancer of the pancreas: is it worthwhile?. J. Gastrointest. Surg..

[73] Allendorf J.D., Lauerman M., Bill A., DiGiorgi M., Goetz N., Vakiani E., Remotti H., Schrope B., Sherman W., Hall M., Fine R.L., Chabot J.A. (2008). Neoadjuvant chemotherapy and radiation for patients with locally unresectable pancreatic adenocarcinoma: feasibility, efficacy, and survival. J. Gastrointest. Surg..

[74] Cheng T.Y., Sheth K., White R.R., Ueno T., Hung C.F., Clary B.M., Pappas T.N., Tyler D.S. (2006). Effect of neoadjuvant chemoradiation on operative mortality and morbidity for pancreaticoduodenectomy. Ann. Surg. Oncol..

[75] Massucco P., Capussotti L., Magnino A., Sperti E., Gatti M., Muratore A., Sgotto E., Gabriele P., Aglietta M. (2006). Pancreatic resections after chemoradiotherapy for locally advanced ductal adenocarcinoma: analysis of perioperative outcome and survival. Ann. Surg. Oncol..

[76] Gillen S., Schuster T., Meyer Zum Büschenfelde C., Friess H., Kleeff J. (2010). Preoperative/neoadjuvant therapy in pancreatic cancer: a systematic review and meta-analysis of response and resection percentages. PLoS Med..

[77] Sperti C., Pasquali C., Bissoli S., Chierichetti F., Liessi G., Pedrazzoli S. (2010). Tumor relapse after pancreatic cancer resection is detected earlier by 18-FDG PET than by CT. J. Gastrointest. Surg..

[78] Okamoto K., Koyama I., Miyazawa M., Toshimitsu Y., Aikawa M., Okada K., Imabayashi E., Matsuda H. (2010). Preoperative 18[F]-fluorodeoxyglucose positron emission tomography/computed tomography predicts early recurrence after pancreatic cancer resection. Int. J. Clin. Oncol..

[79] Meyers M.O., Meszoely I.M., Hoffman J.P., Watson J.C., Ross E., Eisenberg B.L. (2004). Is reporting of recurrence data important in pancreatic cancer?. Ann. Surg. Oncol..

[80] Shibata K., Matsumoto T., Yada K., Sasaki A., Ohta M., Kitano S. (2005). Factors predicting recurrence after resection of pancreatic ductal carcinoma. Pancreas.

[81] Kleeff J., Reiser C., Hinz U., Bachmann J., Debus J., Jaeger D., Friess H., Büchler M.W. (2007). Surgery for recurrent pancreatic ductal adenocarcinoma. Ann. Surg..

[82] Gumbs A.A., Rodriguez Rivera A.M., Milone L., Hoffman J.P. (2011). Laparoscopic Pancreatoduodenectomy: A Review of 285 Published Cases. Ann. Surg. Oncol..

[83] Kooby D.A., Chu C.K. (2010). Laparoscopic management of pancreatic malignancies. Surg. Clin. North. Am..

[84] Nakeeb A. (2009). Laparoscopic pancreatic resections. Adv. Surg..

[85] Briggs C.D., Mann CD, Irving G.R., Neal C.P., Peterson M., Cameron I.C., Berry D.P. (2009). Systematic review of minimally invasive pancreatic resection. J. Gastrointest. Surg..

[86] Kooby D.A. (2008). Laparoscopic pancreatic resection for cancer. Expert. Rev. Anticancer. Ther..

[87] Michalski C.W., Weitz J., Büchler M.W. (2007). Surgery insight: surgical management of pancreatic cancer. Nat. Clin. Pract. Oncol..

[88] Takaori K., Tanigawa N. (2007). Laparoscopic pancreatic resection: The past, present, and future. Surg. Today.

[89] Kooby D.A., Gillespie T., Bentrem D., Nakeeb A., Schmidt M.C., Merchant N.B., Parikh A.A., Martin R.C., Scoggins C.R., Ahmad S., Kim H.J., Park J., Johnston F., Strouch M.J., Menze A., Rymer J., McClaine R., Strasberg S.M., Talamonti M.S., Staley C.A., McMasters K.M., Lowy A.M., Byrd-Sellers J., Wood W.C., Hawkins W.G. (2008). Left-sided pancreatectomy: A multicenter comparison of laparoscopic and open approaches. Ann. Surg..

[90] Mabrut J.Y., Fernandez-Cruz L., Azagra J.S., Bassi C., Delvaux G., Weerts J., Fabre J.M., Boulez J., Baulieux J., Peix J.L., Gigot J.F. (2005). Hepatobiliary and Pancreatic Section (HBPS) of the Royal Belgian Society of Surgery; Belgian Group for Endoscopic Surgery (BGES); Club Coelio. Laparoscopic pancreatic resection: results of a multicenter European study of 127 patients. Surgery.

[91] Ohtsuka T., Tanaka M., Miyazaki K. (2006). Gastrointestinal function and quality of life after pylorus-preserving pancreatoduodenectomy. J. Hepatobiliary Pancreat. Surg..

[92] Fitzsimmons D., Johnson C.D. (1998). Quality of life after treatment of pancreatic cancer. Langenbecks Arch. Surg..

[93] Billings B.J., Christein J.D., Harmsen W.S., Harrington J.R., Chari S.T., Que F.G., Farnell M.B., Nagorney D.M., Sarr M.G. (2005). Quality-of-life after total pancreatectomy: Is it really that bad on long-term follow-up?. J. Gastrointest. Surg..

[94] Wenger F.A., Jacobi C.A., Haubold K., Zieren H.U., Muller J.M. (1999). Gastrointestinal quality of life after duodenopancreatectomy in pancreatic carcinoma. Preliminary results of a prospective randomized study: Pancreatoduodenectomy or pylorus-preserving pancreatoduodenectomy. Chirurg.

[95] Bloechle C, Broering DC, Latuske C. (1999). Prospective randomized study to evaluate quality of life after partial pancreatoduodenectomy according to Whipple versus pylorus preserving pancreatoduodenectomy according to Longmire-Traverso for periampullary carcinoma. Deutsche Gesellschaft für Chirurgie..

[96] Seiler C.A., Wagner M., Bachmann T., Redaelli C.A., Schmied B., Uhl W., Friess H., Buchler M.W. (2005). Randomized clinical trial of pylorus-preserving duodenopancreatectomy versus classical Whipple resection - long term results. Br. J. Surg..

[97] Paquet K.-J. (1998). Comparison of Whipple's pancreaticoduodenectomy with the pylorus- preserving pancreaticoduodenectomy—A prospectively controlled, randomized long-term trial. Chirurgische Gastroenterologie.

[98] Tran K.T., Smeenk H.G., van Eijck C.H., Kazemier G., Hop W.C., Greve J.W., Terpstra O.T., Zijlstra J.A., Klinkert P., Jeekel H. (2004). Pylorus preserving pancreaticoduodenectomy versus standard Whipple procedure: A prospective, randomized, multicenter analysis of 170 patients with pancreatic and periampullary tumors. Ann Surg..

[99] Lin P.W., Shan Y.S., Lin Y.J., Hung C.J. (2005). Pancreaticoduodenectomy for pancreatic head cancer: PPPD versus Whipple procedure. Hepatogastroenterology.

